# Porous anodic alumina on galvanically grown PtSi layer for application in template-assisted Si nanowire growth

**DOI:** 10.1186/1556-276X-6-414

**Published:** 2011-06-08

**Authors:** Irini Michelakaki, Androula G Nassiopoulou, Eleni Stavrinidou, Katerina Breza, Nikos Frangis

**Affiliations:** 1 Institute of Microelectronics, NCSR Demokritos, Terma Patriarchou Grigoriou, Aghia Paraskevi, 153 10, Athens, Greece; 2 Solid State Physics Section, Department of Physics, Aristotle University of Thessaloniki, 54124 Thessaloniki, Greece

## Abstract

We report on the fabrication and morphology/structural characterization of a porous anodic alumina (PAA)/PtSi nano-template for use as matrix in template-assisted Si nanowire growth on a Si substrate. The PtSi layer was formed by electroless deposition from an aqueous solution containing the metal salt and HF, while the PAA membrane by anodizing an Al film deposited on the PtSi layer. The morphology and structure of the PtSi layer and of the alumina membrane on top were studied by Scanning and High Resolution Transmission Electron Microscopies (SEM, HRTEM). Cross sectional HRTEM images combined with electron diffraction (ED) were used to characterize the different interfaces between Si, PtSi and porous anodic alumina.

## Introduction

Semiconductor nanowires (NWs) constitute a fundamental building block for the development of nanoscale devices such as nanowire field effect transistors (FETs), energy harvesting devices, third generation solar cells, sensors and photonic devices. Among them, Si NWs are particularly investigated and a lot of interesting devices based on them have been already demonstrated [1-8]. Compound semiconductor NWs are also intensively investigated for their applications in light emitting devices and lasers [9-11].

One of the most commonly used NW synthesis techniques for both Si and compound semiconductor nanowires is chemical vapor deposition (CVD) using a noble metal as catalyst. The growth follows in general the vapor-liquid-solid (VLS) process. Au is typically used as catalyst, however it is well known that this material, when incorporated into the Si lattice, can introduce deep-level traps in the Si bandgap that are detrimental to any electronic device. As an alternative to Au, Pt is less poisonous to Si electronics and both Pt and PtSi are used as contact metals for Si devices. The growth of crystalline Si nanowires using PtSi as a microelectronics-friendly solid phase catalyst has been demonstrated by Baron et al [12]. In their work, PtSi was formed by physical vapor deposition of Pt followed by thermal annealing at high temperature. The overall objective of the present work is to use low cost fabrication processing to fabricate a nano-template of PAA/PtSi for the growth of ordered nanowires on Si catalysed by the PtSi at the pore bottom of the PAA film. Ordering and controlled positioning of NWs on Si is particularly challenging towards the fabrication of NW-based nanoscale devices. Self-assembled PAA films directly grown on Si by electrochemical oxidation of an Al film [13-18] have received significant attention as a low-cost large-area, controllable pore size and reliable fabrication template for the synthesis of NWs on the Si substrate. PAA pore diameters range from nanometers to sub-micrometers depending on the electrochemical solution and anodization voltage used for their fabrication. Moreover, pore density is much higher compared to other nano-template materials such as polysterene membranes used in nanosphere lithography. PAA pore density can exceed 10^11 ^pores/cm^2 ^[19].

In the following we will report on the galvanic growth of thin PtSi layers on (100) Si at a temperature of 8°C and the subsequent growth of a porous anodic alumina (PAA) membrane on top, that constitutes an excellent low cost reliable fabrication nano-template stack for application in directed Si NW synthesis within the pores. The fabrication processing will be described. Characterization results of the morphology and structure of the PtSi layers with the PAA membrane on top, as well as the different interfaces involved using field emission SEM (FE-SEM), HRTEM and electron diffraction will be presented.

## Experimental results

The substrates used were p-type (100) Si wafers with resistivity 1-2 Ωcm. Prior to Pt deposition, the wafers were chemically cleaned using piranha cleaning followed by an HF dip, rinsing in aceton, isopropanol and deionized water and drying in nitrogen gas flow. When removed from the solution, the samples were again rinsed in deionized water and dried in nitrogen gas flow.

We first investigated the conditions of formation of a PtSi film on Si through galvanic deposition and we then studied the formation of a porous anodic alumina thin film on top of the thin PtSi layer.

### A) Galvanic deposition of PtSi on Si

The galvanic deposition of a noble metal on Si from a solution containing the metal salt was investigated in detail recently [20]. A number of metals including Au, Pt, Ag, Cu and Ni were successfully deposited in the form of metal clusters on Si. As a general condition, the substrate has to show a reduction potential less than that of the metal. Under such conditions, the deposition of the metal is spontaneously favored at the expense of the substrate, which in turn is oxidized. An agent that can remove the oxide during deposition is also needed, otherwise the formed oxide would passivate the substrate surface and prevent further metal deposition. For galvanic deposition on Si the oxide is removed by using HF in the solution. Pt deposition on Si was thus demonstrated by using an aqueous solution of K_2_PtCl_6 _salt with 0.8 mM Pt concentration ([Pt]) in which 48% HF is added at different ratios. The overall reaction which takes place is the following:

In order to get soluble silicon hexafluorite in the solution the molar ratio of HF:Pt ions has to exceed 6:2 [20]. In this work we report on results obtained by using the above Pt salt solution in HF with a concentration ratio [HF]/[Pt] equal to 60 and a solution temperature of 8°. We show that thin layers of PtSi are formed on Si for small immersion times, while for longer immersion times Pt starts to be deposited, forming clusters on the PtSi film. We investigated in detail the structure and morphology of the obtained films using different immersion times, ranging from 5 minutes to 50 minutes. The corresponding samples are denoted in the following by S-5 min, S-15 min, S-30 min and S-50 min.

**Figure 1:**
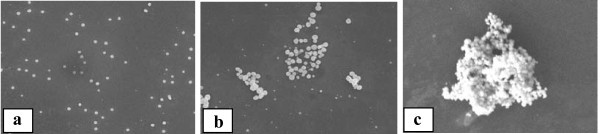
**SEM images of the surface of samples S-15 min (a), S-30 min (b), and S-50 min (c)**. In (a), the surface is smooth and contains a small density of spherical nanoparticles on it, while in (b), we reveal that these nanoparticles started to form clusters, which increase in volume with time (see (c)).

Figure 1 shows FE-SEM images of samples S-15 min (a), S-30 min (b) and S-50 min (c). The surface film morphology is smooth in all cases with a number of dots or clusters of dots superimposed on it. These dots/clusters were not observed in sample S-5 min (not shown in Figure 1 since its surface was totally featureless). In sample S-15 min the surface contains only a small density of spherical dots, which tend to merge progressively into clusters of dots as the immersion time increases (see samples S-30 min (Figure 1b) and S-50 min (Figure 1c)).

**Figure 2:**
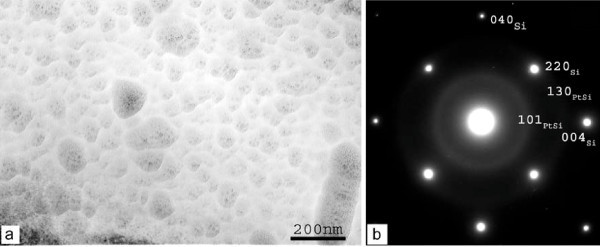
**Plane-view bright-field image (a) and the corresponding electron diffraction pattern (b) from sample S-5 min**. The rings in the diffraction pattern correspond to Si and PtSi structures. No Pt nanocrystals were detected.

**Figure 3:**
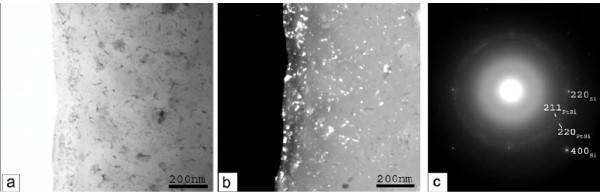
**Plane-view bright-field image (a), dark-field image (b), and the corresponding electron diffraction pattern (c) from sample S-15 min**. Rings from Si (substrate) and PtSi structures are revealed in the diffraction pattern. The spherical nanoparticles on the surface (images (a) and (b)) are relatively small and well separated between them.

**Figure 4:**
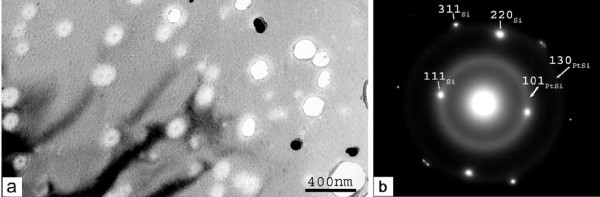
**Plane-view bright-field image (a) and the corresponding electron diffraction pattern (b) from sample S-30 min**. Si and PtSi rings are revealed, while no Pt rings are detected. The nanofeatures on the plane-view image seem to be holes corresponding to the footprint of nanocrystals that were probably removed during sample preparation. Their diameter is larger than the PtSi nanoparticles observed in sample S-15 min.

TEM images combined with the corresponding diffraction patterns depicted that:

a) The S-5 min film was amorphous and homogeneous in morphology with a limited surface roughness. The corresponding diffraction pattern showed diffuse rings corresponding to PtSi. These results are illustrated in Figure 2(a, b). In (a) a bright field image is shown, while in (b) the corresponding electron diffraction pattern is depicted. The rings in the diffraction pattern correspond to Si and PtSi, while no Pt nanocrystals were detected.

b) The S-15 min sample surface was covered by a small number of nanocrystals of sizes ranging from 10 to 100 nm and lying on an amorphous layer. The nanocrystals were identified as PtSi nanocrystals, as shown in the corresponding diffraction pattern and dark field TEM images of Figure 3. Figure 3a shows a plane view bright field image, Figure 3b a dark field image and Figure 3c the corresponding electron diffraction pattern. Rings from Si (substrate) and PtSi structures are revealed in the diffraction pattern. The spherical nanoparticles on the surface (images (a) and (b)) are relatively small and well separated between them.

c) The S-30 min sample showed a large number of holes on an amorhous PtSi film (see bright field TEM image (Figure 4a) and the corresponding diffraction pattern (Figure 4)). The holes are probably the footprint of nanocrystals and clusters of nanocrystals, as those identified in the corresponding SEM images, that were removed during TEM sample preparation. Their diameter was larger than the nanoparticles of sample S-15 min.

From the above results it is clear that a PtSi film is formed on the Si surface during the first minutes of immersion of the sample into the Pt salt used (with an [HF]/[Pt] ratio of 60). As the immersion time is increased, PtSi nanocrystals start to form, which merge progressively into clusters of nanocrystals. The deposition of Pt on top of the PtSi layer was observed at longer immersion times.

### Porous anodic alumina template on the galvanically deposited PtSi layer

**Figure 5:**
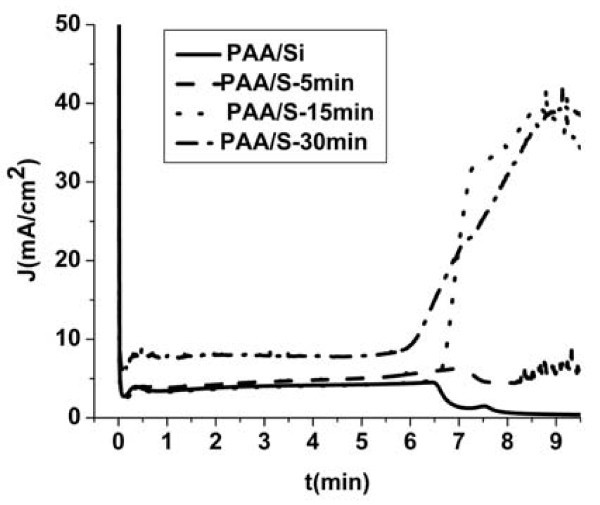
**Current-time anodization curves of an Al film on Si and on different PtSi/Si substrates (samples S-5 min, S-15 min, and S-30 min)**. In all cases, the Al film thickness was 500 nm. The anodization curve of sample Al/S-5 min is quite similar to that of Al/Si, while in the cases of samples Al/S-15 min and Al/S-30 min the current increases significantly when the PAA film reaches the underlying surface.

Porous anodic alumina (PAA) can be grown on Si by anodic oxidation of an Al film. In this work, we investigated the formation of a PAA template on top of the PtSi layer with the objective to use the PtSi layer underneath as a catalyst for the formation of Si nanowires into the alumina pores. We used three of the PtSi films grown above, namely S-5 min, S-15 nm and S-30 min. An Al film, 500 nm thick, was deposited on the PtSi/Si substrate and an Al ohmic contact was formed on the backside of the wafer. The samples were then anodized in sulfuric acid aqueous solution at an anodization voltage of 20 V. The corresponding current-time anodization curves, compared to the one obtained without the PtSi layer, are shown in Figure 5. The general form of the curves is the same as that of the Al/Si anodization curve up to the time that the pores reach the Al_2_O_3_/Si or Al_2_O_3_/PtSi interface. The different phases of the anodization process are as follows [21]: At the very beginning of the anodization the current drops spontaneously to a lower value due to initiation of the oxidation of the Al surface. It then starts to increase with a certain rate during the phase of initiation of pore formation and it almost stabilizes when pore formation is on-going. This phase continues until the pores reach the interface with Si and it then drops suddenly to a minimum value. This current decrease corresponds to the initiation of oxidation of the Si surface underneath the PAA film [21]. The anodization curve of the sample PAA/S-5 min followed the same behavior with that described above, which means that a thin SiO_2 _film was formed underneath the PtSi layer by oxygen diffusion through the silicide. On the other hand, in the other two cases of samples PAA/S-15 and PAA/S-30 an abrupt increase of the current density with time was observed at the end of Al oxidation. As it will be illustrated below in the TEM images, this is due to the fact that the pore bottom reaches the surface of metal dots present on the PtSi film in these two last samples and an important leakage current flows to the Si substrate through these dots.

Cross sectional TEM images were obtained from two different samples to illustrate the above results. In the first case the sample used was PAA/S-15 min and the process was stopped before the final current increase in the anodization curve. In the second case the sample PAA/S-30 min was used and the process continued for some time after the current increase initiation. The corresponding cross sectional TEM image and electron diffraction pattern showed the following behavior:

Sample PAA/S-15 min, end of process before the final abrupt increase in the anodization current

**Figure 6:**
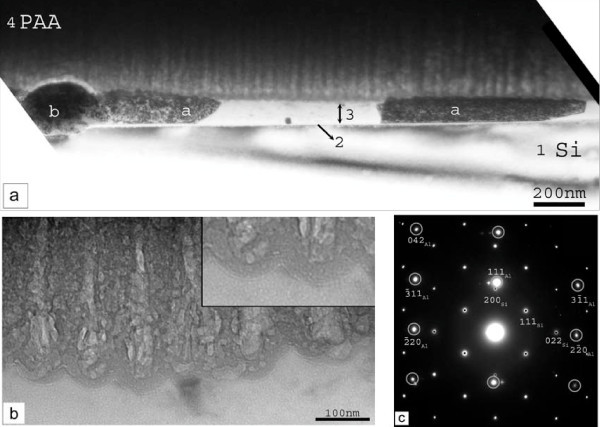
**Cross-sectional bright-field TEM images (panels (a), (b)) and the corresponding electron diffraction pattern (panel (c)) (sample PAA/S-15 min/Si)**. In this sample the anodization was stopped before the abrupt current increase in the anodization current. In panel (a), we reveal an amorphous layer (indicated by 2) on the Si substrate (indicated by 1). On top of this thin amorphous layer, there is another layer (layer 3) in which we identify different round-shaped multi-phase clusters of Pt-Al-Si (indicated by b) and Al nanocrystals (indicated by a). In panel (b), the pores of the PAA film are shown in larger magnification, while the inset illustrates the barrier layer at the bottom of the pores. The electron diffraction pattern of panel (c) reveals that some of the Al nanocrystals are in very good epitaxial relationship with the Si substrate.

The corresponding results are shown in Figure 6. In (a) and (b) we see the cross sectional bright field TEM images, while in (c) the corresponding electron diffraction pattern. In panel (a) we reveal the Si substrate (indicated by 1), a very thin amorphous layer (indicated by 2) and another layer on top (indicated by 3). Within this last layer we identify Al nanocrystals (indicated by (a)) and round shape multi-phase clusters, containing different phases of Pt, Al and Si. This means that in this sample the Al film is not fully transformed into Al_2_O_3_, but some Al nanocrystals still remain. From the electron diffraction pattern of panel (c) it was revealed that some of the Al nanocrystals are in very good epitaxial relationship with the Si substrate. In panel (b) the pores of the porous anodic alumina (PAA) are shown in larger magnification, while the inset illustrates the barrier layer at the bottom of the pores.

Sample PAA/S-30 min, end of the process after the final abrupt increase in the anodization current

**Figure 7:**
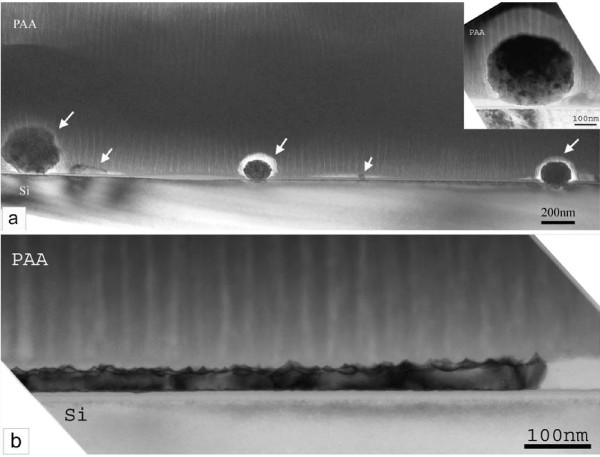
**Cross sectional bright field images of sample S-30min with a PAA film on top. **In panel  (a) the morphology of the interface with the Si substrate  is shown, depicting the presence of large nanograins between the PAA film and Si (attributed to Pt nanograins and illustrated in higher magnification in the inset of panel (a)). On top of these nanograins the alumina barrier layer usually observed at each pore bottom at the interface of PAA films with Si (in the absence of Pt in-between) is missing. Panel (b) shows another part of the sample cross section which illustrates that occasionally a continuous film of crystalline Pt   exists between the PAA film and the Si substrate. The white area on the right depicts either amorphous parts of this film or Pt crystals with different orientation.

The corresponding results are shown in Figure 7. At the interface of Pt with Si large nanograins are depicted, as those identified on the surface of sample S-30 min (see panel a). The barrier layer usually observed at each pore bottom is absent above the nanograin and the pores seem to reach directly the grain surface. This explains the appearance of the high leakage current in the anodization curve, which can be attributed to a direct current flow from the electrolyte to the Si surface through the nanograin. The existence of the grains prevents the homogeneous oxidation of the Si surface through the pores at the Al_2_O_3_/Si interface.

## Conclusions

The formation of a PAA/PtSi nano-template on Si by galvanic deposition of Pt, physical vapor deposition of Al and anodic oxidation of the Al film was investigated in detail. Depending on the immersion time of the samples into the Pt salt solution, a thin PtSi layer on Si can be obtained, on top of which a homogeneous PAA template can be formed. This nano-template is very appropriate for the growth of ordered Si nanowires within the pores using the VLS technique catalyzed by the PtSi nanofilm at the bottom of each pore.

## Competing interests

The authors declare that they have no competing interests.

## Authors' contributions

IM performed sample preparation, AGN supervised the work and wrote the paper, ES, AB and NF performed the TEM work.
